# Impact of cerebellar stroke on established and emergent reading skills: Evidence of alexia

**DOI:** 10.21203/rs.3.rs-6614964/v1

**Published:** 2025-05-16

**Authors:** Anna Chrabaszcz, Julie A. Fiez

**Affiliations:** University of Pittsburgh; University of Pittsburgh

**Keywords:** cerebellum, stroke, reading, alexia

## Abstract

This study examined the impact of cerebellar stroke on both well-established reading skills and emergent literacy abilities using a combination of static and dynamic assessments. The static component involved a lexical decision task featuring novel orthographic forms (pseudowords) followed by tests of orthographic memory. The dynamic component employed a training protocol in an artificial orthography, requiring participants to learn new grapheme-phoneme correspondences and read in a novel script. Participants included individuals with cerebellar stroke (n = 13) and demographically matched controls (n = 13). Results indicated that cerebellar damage impairs phonological decoding processes, disrupting both reading of novel forms in a familiar orthography and the acquisition of new orthographic-phonological mappings. Notably, five of the 13 cerebellar patients met criteria for phonological alexia, though no clear relationship emerged between symptom severity and lesion characteristics. These findings underscore the cerebellum’s role in phonological decoding and its contribution to both established and emergent aspects of reading.

## Introduction

Our understanding of the cerebellum has undergone a radical shift over the past few decades, expanding beyond its traditional role in motor control to encompass non-motor cognitive functions (Konczak & Timmann, 2007; Schmahmann, 2019; Strick, Dum, & Fiez, 2009; Vlasova, Panikratova, & Pechenkova, 2023). One cognitive domain where the relevance of the cerebellum has been noted is reading and orthographic processing (De Smet et al., 2007; Mariën et al., 2014; Mariën, P., & Beaton, 2014; Stoodley, 2012; Vlachos, Papathanasiou, & Andreou, 2007). Support for this role comes from neuroimaging studies of typical readers and individuals with dyslexia, as well as clinical investigations of patients with cerebellar damage due to stroke, tumors, and degeneration.

While the cerebellum is not traditionally considered a core component of the reading network, neuroimaging studies consistently report robust cerebellar activation during tasks involving written stimuli, including characters, real words, pseudowords, and sentences (e.g., Christodoulou et al., 2014; D’Mello et al., 2020; Fiez & Petersen, 1998; Fulbright et al., 1999; Lesage, Hansen, & Miall, 2017; Li et al., 2021; Li, Yuan, Luo, & Tao, 2022; Mechelli et al., 2003; Turkeltaub et al., 2002). Reading-related cerebellar activation is most commonly observed in lobules VI–VIIb and Crus I–II, often with a right-hemisphere dominance (D’Mello et al., 2020; Li, Yuan, Luo, & Tao, 2022; Stoodley, 2012; Stoodley & Stein, 2011). These regions exhibit functional connectivity with cerebral areas involved in reading, including the inferior frontal, temporo-parietal, and occipito-temporal cortices (Alvarez & Fiez, 2018; Stoodley, 2012; Strick, Dum, & Fiez, 2009). For example, right lobule VIIb and Crus II are functionally connected to the inferior frontal junction and superior parietal lobule during phonological processing, while right Crus I–II exhibits connectivity with the middle temporal gyrus during lexical-semantic processing (Alvarez & Fiez, 2018; Li et al., 2022; Li, Yuan, Luo, & Tao, 2022). These findings support the notion of a coordinated cerebro-cerebellar system, with the cerebellum contributing to phonological, orthographic, and semantic aspects of reading.

Evidence for cerebellar involvement in reading is also provided by studies on dyslexia, showing that individuals with dyslexia exhibit distinct patterns of cerebellar activation (Baillieux et al., 2009; Beneventi et al., 2010; Nicolson et al., 1999) and have reduced cerebellar gray matter compared to typical readers (Brambati et al., 2004; Brown et al., 2001; Pernet et al., 2009). Moreover, dyslexic children often display motor coordination deficits, balance impairments, poor handwriting, and slower processing speed—symptoms consistent with cerebellar dysfunction (Fawcett & Nicolson, 1999; Fawcett, Nicolson, & Dean, 1996). These findings have contributed to the development of the “cerebellar deficit hypothesis” (Nicolson et al., 1999; Nicolson et al., 2001), which posits that cerebellar abnormalities present from birth disrupt motor control and articulation, leading to deficits in phonological representations and impairments in the phonological loop of working memory. These deficits, in turn, interfere with the development of reading, writing, and spelling skills.

The link between the cerebellum and reading is further evidenced by neuropsychological assessments of patients with cerebellar damage due to stroke or tumor resection. Some patients experience difficulties with reading sentences (Fabbro, Moretti, & Bava, 2000), nonwords (Mariën et al., 2009; Paul, Baca, & Fischer-Baum, 2022), and words presented in non-standard orientations, such as mirror-reversed or upside-down (Paul, Baca, & Fischer-Baum, 2022). Additionally, deficits in reading comprehension, including understanding of words, sentences, and short stories, have also been documented (Fabbro et al., 2004; Fabbro, Moretti, & Bava, 2000; Geva et al., 2021; Karacı et al., 2008; Mariën et al., 2009; Savaş et al., 2024). Corroborating evidence from eye-tracking studies indicates that survivors of cerebellar tumors exhibit slower reading times, a greater number of total fixations, and increased saccadic regressions compared to control participants (Mironets, Shurupova, & Dreneva, 2022).

Although these clinical findings offer important insights into the cerebellum’s role in reading, they have typically emerged from broader neuropsychological batteries rather than targeted, systematic investigations of reading processes. To date, only two studies have directly examined the causal impact of cerebellar lesions on reading. One such study by Moretti et al. (2002) found that patients with (para)vermian cerebellar lesions exhibited greater difficulty reading words, nonwords, sentences, and passages than control participants, providing first evidence of acquired reading difficulties (i.e., alexia) due to cerebellar damage. Another study by Ben-Yehudah and Fiez (2008) evaluated reading skills and phonological processing in six patients with focal damage in the left and right cerebellum. While reading comprehension, accuracy and fluency did not differ from those of matched healthy controls, performance on a visual rhyme judgement task was compromised, particularly in patients with anterior/superior cerebellar lesions, especially when items shared orthography but not phonology (e.g., fear-bear), a pattern also observed in individuals with dyslexia. Patients also had difficulty recalling lists of nonwords relative to real words. Given that rhyme judgement and serial recall of nonwords engage verbal working memory resources and subvocal rehearsal, the authors attributed these deficits to impaired articulatory monitoring due to cerebellar damage.

Taken together, neuropsychological findings suggest that cerebellar dysfunction contributes to reading deficits. However, conclusions are often drawn from small sample sizes (e.g., one patient in Marien et al. (2009) and Paul, Baca, & Fischer-Baum (2022); two patients in Fabbro et al. (2004); four patients in Fabbro, Moretti, & Bava (2000) and Geva et al. (2021); six patients in Ben-Yehudah & Fiez (2008)) and, in some cases, lack appropriate control comparisons. These limitations hinder the generalizability of findings and underscore the need for further research to delineate the cerebellum’s specific contributions to reading. Moreover, prior research has assessed patients’ reading ability statically, that is, by measuring pre-existing, established literacy skills without considering the capacity to acquire new ones. This approach overlooks the cerebellum’s well-documented role in learning and adaptation, particularly in implicit learning tasks (Doyon, Penhune, & Ungerleider, 2003; Matsumura et al., 2004; Timmann et al., 2010).

The present study addresses these limitations by combining a conventional static approach with an innovative dynamic assessment approach to evaluate participants’ ability to learn new reading skills given practice and feedback (Dixon, Oxley, Gellert, & Nash, 2023; Dixon, Oxley, Nash, & Gellert, 2023; Grigorenko, 2009). Unlike static assessment, which reflects accumulated reading experience, dynamic assessment captures latent learning potential while minimizing biases related to socioeconomic background and prior literacy experiences and opportunities (Peña, Iglesias, & Lidz, 2001).

For static assessment of participants’ established reading skills in their native language (English), we administered a lexical decision task (LDT) with a reading-aloud component and an orthographic learning assessment. The LDT enables comparison of reading performance for real words versus pseudowords, while the orthographic learning assessment evaluates participants’ recognition and recall of unfamiliar word forms (pseudowords) with a choice and a spelling task, respectively. Additionally, we administered the Word Identification and Word Attack subtests of the Woodcock Reading Mastery Test (WRMT; Woodcock, 2011)—to establish a standardized, norm-referenced reading performance of words and pseudowords, respectively.

Our dynamic assessment approach considers that learning to read, at least in alphabetic languages, relies heavily on phonological decoding, which hinges on the ability to convert arbitrary visual symbols into speech sounds to derive meaning (Castles, Rastle, & Nation, 2018; Rozin & Gleitman, 1977; Share, 1995). It is a foundational process in emergent reading and serves as a critical step toward reading proficiency by reinforcing the connections between orthography, phonology, and meaning (Share, 2008). To examine such emergent reading skills in the context of dynamic assessment, we developed an artificial orthography (AO) learning paradigm, allowing us to investigate how individuals with cerebellar damage acquire novel letter-sound associations and apply phonological decoding strategies to read words in an unfamiliar script. Of note, robust cerebellar activation has been previously observed in neuroimaging studies with artificial scripts (Bitan et al., 2005; AUTHORS; Hashimoto & Sakai, 2004; Quinn, Taylor, & Davis, 2017), pointing towards cerebellar involvement in learning new literacy skills in the context of artificial reading paradigms.

Using this innovative methodological approach, the goal of this study is to determine whether the impact of stroke-related cerebellar damage affects established reading skills, emergent reading skills, or both. Additionally, given the cerebellum’s role in phonological processing and learning (Ben-Yehudah & Fiez, 2008; Nicolson et al., 1999, 2001), we hypothesize that cerebellar stroke participants will exhibit greater impairments in learning letter-sound correspondences and poorer performance on the tasks with a strong emphasis on phonological decoding skills, such as pseudoword and AO reading. Impaired performance on these tasks will be considered a manifestation of acquired phonological alexia (Beauvois & Derouesne, 1979; Patterson & Ralph, 1999; Tree & Kay, 2006). Overall, by integrating dynamic assessment with traditional reading measures, our study aims to provide a more comprehensive understanding of the cerebellum’s contributions to reading, orthographic learning, and reading development.

## Method

### Participants

Thirteen patients with cerebellar lesions due to stroke and 13 neurologically healthy controls participated in this study. The patient and control groups were closely matched for gender (5 female, 8 male participants in each group), race (patients: 10 white, 3 Black/Afro-American; controls: 11 white, 2 Black/Afro-American), age (patients: *M* = 64.8, *SD* = 11.4; controls: *M* = 65.7, *SD* = 11.7), and years of education (patients: *M* = 13.9, *SD* = 1.9; controls: *M* = 13.6, *SD* = 1.6). All participants were right-handed, native speakers of American English, with no history of neurocognitive or psychiatric disorders.

**Figure 1:**
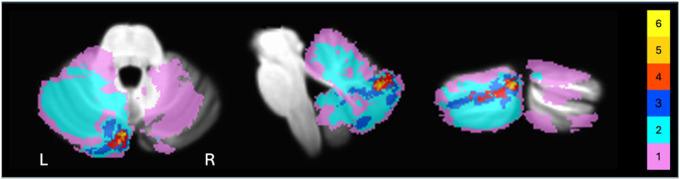
Cerebellar lesion overlay in the stroke patient group. Note. The color indicates the number of patients with damage to a given voxel. The greatest overlap (yellow) corresponds to Left Crus II and includes lesions from six subjects.

In the patient group, seven people had lesions in the left cerebellar hemisphere, three in the right hemisphere, and three had bilateral lesions. Maximum lesion overlap (n = 6) occurred in Left Crus II (Fig. 1). Twelve patients had ischemic strokes; one patient had a hemorrhagic stroke. The time between stroke occurrence and testing ranged from 5 months to 13.5 years (*M* = 5.17, *SD* = 4.22). There was considerable variability in cerebellar lesion sizes, ranging from 415 to 74,849 mm^3^. None of the patients presented with gross motor speech deficits, as assessed by the Frenchay Dysarthria Battery (Enderby, 1980).

All participants provided informed consent and were compensated for their time. All procedures were performed in accordance with the ethical principles for conducting research on human subjects outlined in the Declaration of Helsinki and were approved by the Institutional Review Board at the University of Pittsburgh (STUDY19070420).

## Materials

### Neuropsychological measures.

All participants underwent a comprehensive neuropsychological assessment of 1) *motor function*: 8-Meter Walking Test, 9-Hole Peg Test (Schmitz-Hubsch et al., 2008); Finger Tapping Test; and the Scale for the Assessment and Rating of Ataxia (SARA) (Schmitz-Hubsch et al., 2006); 2) *cognitive and executive function*: Montreal Cognitive Assessment scale (MoCA) (Nasreddine et al., 2005); Rey Auditory-Verbal Learning Test (RAVLT) (Rey, 1983); and Color-Word Interference subtest of the Delis-Kaplan Executive Function System (D–KEFS) (Delis, Kaplan, & Kramer, 2001); and 3) *reading function*: the WRMT’s Word Identification and Word Attack subtests (Woodcock, 2011); Rapid Letter Naming subtest of the Comprehensive Test of Phonological Processing (CTOPP) (Wagner et al., 2013). Scores from the Word Identification (real word reading) and Word Attack (pseudoword reading) subtests constituted part of the static assessment and established norm-referenced reading performance of words and pseudowords.

### English stimuli.

Sixteen monosyllabic target pseudowords, each 4 to 6 letters in length (e.g., “bleaz,” “nurch”) and 32 real English words with a substantial orthographic overlap with the target pseudowords (e.g., “bleak”, “lurch”) were selected for the LDT. The target pseudowords were drawn from the corpus used in AUTHORS (2023, Experiment 2). Additionally, to assess participants’ orthographic memory of the target pseudowords, three sets of distractor pseudowords were created: 16 homophonous pseudowords differing in the vowel letter(s) but sharing the same pronunciation (e.g., “bleez,” “nerch”), 16 pseudoword foils with consonant alterations (e.g., “bleax,” “nurck”), and 16 foils with both consonant and vowel changes (e.g., “bleex,” “nerck”). These stimuli were used in the orthographic choice task. Participants also completed a pseudoword spelling task, for which the 16 target pseudowords were audio recorded by a native speaker of American English. See Appendix Table A1 for the complete list of stimuli.

### Artificial orthography (AO) stimuli.

Participants learned an artificial alphabet consisting of eight symbols that mapped onto three English vowels (/a /, /e /, / /) and five consonants (/k/, /p/, /r/, /s/, /t/). To assess participants’ ability to read words in the newly learned orthography, we created three sets of English monosyllabic words, each 2 to 4 phonemes in length (six words for reading practice, 12 words for reading with feedback, 18 words for reading without feedback) and transliterated them into the artificial alphabet. The words in each set were counterbalanced for the vowel condition and the length of phonemes. Each word in the reading sets (with and without feedback) constituted a minimal pair (e.g., pie-pay) and could potentially be misread if the vowel identity was decoded incorrectly (see Appendix Table A2). The pronunciations of the letters were recorded by a native speaker of American English; the pronunciations of words were synthesized with the google text-to-speech (gTTS) Python library (language: U.S. English).

## Procedure

Tasks were administered in the following order: MoCA, LDT with reading aloud, AO learning and reading, neuropsychological assessment tasks, orthographic memory tasks (choice and spelling). For all participants, the LDT and orthographic memory tasks were separated by a two-hour interval. Stroke patients also underwent imaging scans for subsequent lesion tracing. All computerized tasks were programmed and delivered using the PsychoPy environment (Peirce, 2019).

### Static assessment with English stimuli.

Participants performed an LDT consisting of three blocks of repeated items. Each block contained 16 pseudowords, each presented twice, and 32 real words, with a total of 64 randomized items per block (Appendix Table A1). Before making a lexical decision, participants were instructed to read each item aloud while their responses were recorded. At the beginning of the task, they completed four practice trials (two pseudowords and two real words). Participants were not informed that their memory of the pseudowords would be tested later.

Two hours after completing the LDT, participants performed a choice and a spelling test to evaluate their orthographic memory of the target pseudowords encountered in the LDT. In the choice test, participants were presented with four options on the computer screen and asked to select the target pseudoword. The options included the target pseudoword (e.g., “bleaz”), a homophonous vowel foil (e.g., “bleez”), a consonant foil (e.g., “bleax”), and a consonant + vowel foil (e.g., “bleex”). In the spelling test, participants heard the target pseudoword and were asked to reproduce its spelling by either typing it on the computer or writing it down on paper.

**Figure 2:**
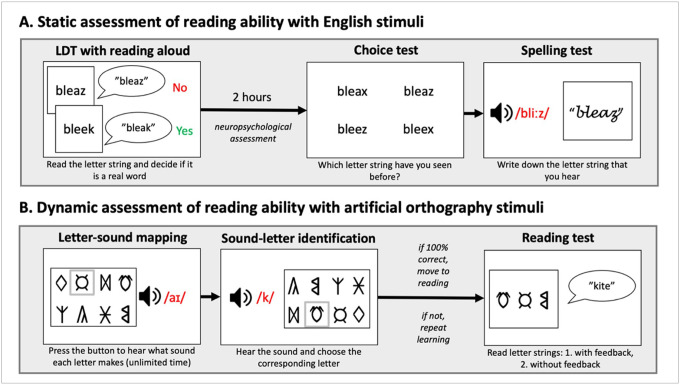
Schematic procedures in A) the static assessment protocol with English stimuli, and B) the dynamic assessment protocol with artificial orthography stimuli.

See Fig. 2A for a schematic of the task procedures.

### Dynamic assessment with AO stimuli.

Participants were informed that they would learn eight new letters, each corresponding to an English speech sound, and use this knowledge to decode English words written in the new script—similar to deciphering a code. They first received an explicit explanation of the letter-sound correspondences through a PowerPoint presentation, which they reviewed together with the experimenter. Following this introduction, participants completed a letter-sound mapping task on the computer. During this task, they could click on a button to hear each letter’s pronunciation. Participants were encouraged to repeat the sounds aloud and replay them as many times as needed until they felt confident to proceed to the sound-letter test (Fig. 2B). In the sound-letter identification task, participants heard a sound and selected the corresponding letter from options displayed on the screen. The position of letters on the screen changed after every button press. In the first block of the task, participants received immediate feedback—if they selected an incorrect letter, the correct letter was displayed. In the second block, participants performed the same task, but without feedback. To advance to the reading task, participants had to achieve 100% accuracy in at least one of the test blocks. If they did not meet this criterion, they repeated the letter-sound training and the sound-letter identification task, with a maximum of three attempts.

The AO reading test consisted of two blocks: with and without feedback. In the feedback block, participants attempted to decode 12 words and heard the correct answer after each response, regardless of their reading accuracy. Words were presented in a fixed order, organized by phoneme length and vowel condition. In the no-feedback block, participants read 18 words without receiving feedback. In this block, word presentation was randomized. Participants’ responses were recorded, and they were given six practice items at the beginning of the reading test.

## Imaging data acquisition and lesion tracing

High-resolution imaging data (T1-weighted, T2-weighted, T2-weighted FLAIR) were acquired with a 64-channel radio frequency head coil on a 3T Siemens Prisma scanner at the Carnegie Mellon University Pitt Brain Imaging Data Generation and Education Center Core Facility (RRID:SCR_023356). All data were collected in sagittal orientation with a resolution of 1-mm isotropic voxel and a 256-mm field of view, ensuring full cerebellar coverage. The parameters were as follows: T1-weighted (208 slices, TR = 2300 ms, TE = 2.03 ms, TI = 900 ms), T2- weighted (176 slices, TR = 3000 ms, TE = 294 ms), and T2- weighted FLAIR (208 slices, TR = 6000 ms, TE = 388 ms, TI = 2200 ms).

To prepare structural images for lesion tracing, the images were first de-obliqued and co-registered using AFNI software (Cox, 1996). The SUIT toolbox (Diedrichsen, 2006; https://diedrichsenlab.org/imaging/suit.htm) was then used to crop out the cerebellum from T1-weighted images. Lesions were manually traced on the cropped T1 images in each participant’s native brain space using ITK-SNAP (Yushkevich & Gerig, 2017), following the guidelines outlined by Lo et al. (2023). Tracing results were verified against T2-weighted and FLAIR images. Lesion tracings were checked by a senior neuroimaging expert. Next, a cerebellum mask was created for each participant in AFNI by combining the cropped cerebellum mask with the lesion mask, filling in missing voxels due to the lesion. This mask was then used to normalize each participant’s cerebellar images to the SUIT template. Finally, the resulting normalization deformation matrix was applied to the lesion mask, brining it into the SUIT template space.

### Analysis

Data analysis was conducted in the R program for statistical computing (version 4.4.1) (R Core Team, 2024). The ‘lme4’ package (Bates et al., 2015) was used to model linear mixed effects (LME) (lmer function) for continuous data and logistic generalized linear mixed effects (GLME) (glmer function) for binary data. The fixed factors included participant group (patients vs. controls), experimental conditions, and the interaction term. They were sum-contrast coded to estimate main effects rather than simple effects. The random-effects structure included varying intercept for participants and items. Post-hoc pairwise comparisons with the Bonferroni adjustment for multiple comparisons were performed with the ‘*emmeans*’ package (Lenth, 2020). Data visualizations were performed with the ‘*ggplot2*’ and ‘*tidyverse*’ packages (Wickham, 2016; Wickham et al., 2019).

## Results

### Neuropsychological assessment

We compared the behavioral performance of patients and control participants across neuropsychological measures using the Welch’s two-sample *t*-test. Significant group differences were observed only in the 9-Hole Peg Test and the D-KEFS Color-Word Interference Test (latency), with patients performing worse than the control group (Table 1). Additionally, patients demonstrated lower reading scores on the Word Identification and Word Attack subtests of the WRMT, suggesting impaired reading of both real English words and pseudowords. Word reading errors in the Word Identification subtest were primarily evident in the low-frequency words like “zeitgeist”, “oeuvre”, etc.

### Static assessment of established reading abilities

#### LDT performance.

Lexical decision accuracy was at ceiling for both groups (patients: *M* = 0.96, *SD* = 0.04, controls: *M* = 0.98, *SD* = 0.03) and was therefore not subjected to inferential statistical analysis.

**Figure 3:**
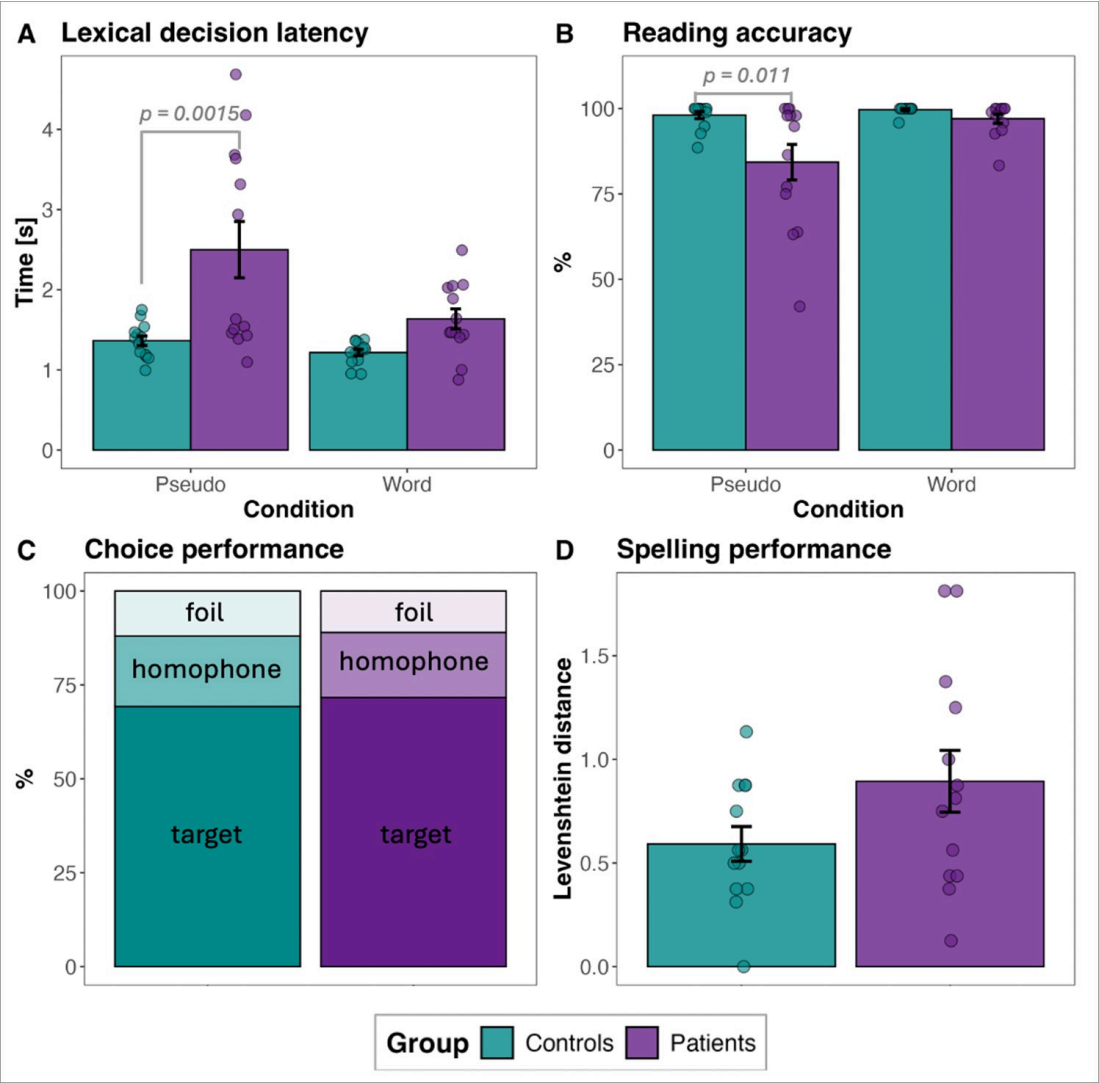
Participants’ performance on the static assessment tasks: A) LDT decision latency, B) LDT reading accuracy, C) choice accuracy, and D) spelling accuracy.

Lexical decision latency data were processed as follows: incorrect trials were excluded, reaction times were log-transformed, and outliers were removed using a threshold of 2.5 absolute deviations around the median for each participant and each condition (Leys et al., 2013). This procedure resulted in the exclusion of 5.2% of the data. An LME model of log-transformed LDT latency data with group (patients vs. controls), condition (words vs. pseudowords), and their interaction as fixed effects, and subjects and items as random effects, revealed a significant effect of group (*β* = −0.18, *SE* = 0.05, *t* = −3.33, *p* = 0.0028), condition (*β* = 0.10, *SE* = 0.01, *t* = 10.2, *p* < 0.001), and interaction (*β* = −0.05, *SE* = 0.004, *t* = −14.22, *p* < 0.001). Post-hoc tests with the Bonferroni adjustment for multiple comparisons indicated that decision latencies were longer for pseudowords than words in both the patient (pseudowords: *M* = 2.5, *SD* = 1.27, words: *M* = 1.64, *SD* = 0.4, *p* < 0.0001) and control (pseudowords: *M* = 1.36, *SD* = 0.22, words: *M* = 1.22, *SD* = 0.15, *p* = 0.0001) groups and that there were latency differences between the two groups in the pseudoword condition (*p* = 0.0015), but not the word condition (Fig. 3A).

We then examined participants’ reading accuracy of words and pseudowords. A GLME model of accuracy data with group, condition, and their interaction as fixed effects, and subjects and items as random effects, revealed a significant effect of group (*β* = −3.59, SE = 1.15, *z* = −3.12, *p* = 0.0018) and condition (*β* = 2.33, *SE* = 0.69, *z* = 3.36, *p* < 0.001), but no significant interaction (*p* = 0.61). Post-hoc tests on log-odds ratios with the Bonferroni adjustment for multiple comparisons indicated that reading accuracy was lower for pseudowords than words in both the patient (pseudowords: *M* = 0.84, *SD* = 0.19, words: *M* = 0.97, *SD* = 0.05, *p* < 0.0001) and control (pseudowords: *M* = 0.98, *SD* = 0.04, words: *M* = 0.997, *SD* = 0.01, *p* = 0.0046) groups and that there was a significant difference between the two groups in pseudoword reading (*p* = 0.011), but a marginal one in word reading (*p* = 0.054) (Fig. 3B). Errors in pseudoword reading were heterogeneous, including lexicalizations (“small” instead of “smeel”), insertions (“fleave” instead of “feave”), vowel alterations (“shrok” instead of “shroak”), consonant alterations (“droap” instead of “broap”), but did not have a clear pattern.

#### Choice test performance.

Cerebellar stroke patients and control subjects chose the target pseudowords more frequently (patients: *M* = 72%, controls: *M* = 69%) than distractor homophone foils or non-homophonous foils, indicating moderate orthographic learning in both groups (Fig. 3C). A chi-square test revealed no significant association between participant group and choice type, *χ*^2^ (2, *N* = 26) = 0.29, *p* = 0.87, suggesting comparable recognition rates for novel orthographic forms in both groups.

#### Spelling test performance.

Pseudoword spelling performance was assessed using Levenshtein distances, which quantify the number of deletions, insertions, and substitutions required to transform a participant’s spelling into the target spelling (with 0 indicating an exact match). Although patients’ spellings deviated more from the target pseudowords’ spellings than those of control participants (patients: *M* = 0.89, *SD* = 0.54, controls: *M* = 0.59, *SD* = 0.3) (Fig. 3D), this difference was not statistically significant, as determined by an LME, *p* = 0.09.

### Dynamic assessment of emergent reading abilities

#### Sound-letter identification.

**Figure 4:**
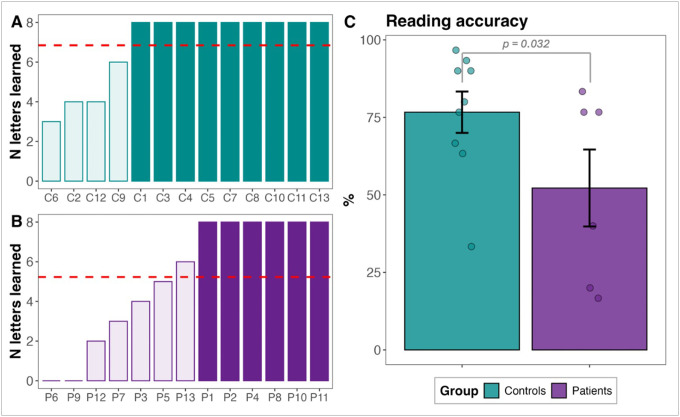
Participants’ performance on the tasks of the artificial orthography training protocol: A) and B) sound-letter identification, and C) reading. Red dashed lines represent group means. A lighter color in panels A and B indicates participants who did not meet the threshold to advance to the reading task.

For each participant, we calculated the maximum number of learned letters across all attempted blocks. Two participants with cerebellar stroke (P6, P9) voluntarily withdrew from the AO protocol during the first training attempt due to difficulty remembering the letter-sound associations; these participants were assigned a score of 0 for the number of learned letters. On average, the patient group learned 5.23 letters, whereas the control group learned 6.87 letters (Fig. 4A, 4B). Participants who correctly identified at least eight letters in one or more task blocks (six patients, nine controls) proceeded to the reading task. We compared reading accuracy of these participants using a GLME model with group as a fixed factor and items and participants as random factors. Results indicated that stroke participants had lower accuracy rates (*M* = 0.52, *SD* = 0.3) compared to control participants (*M* = 0.77, *SD* = 0.2), *β* = 0.68, *SE* = 0.32, *z* = 2.14, *p* = 0.032 (Fig. 4C).

### Individual differences in the cerebellar stroke group

To examine the relationship between deficits of the established reading ability and emergent reading ability in cerebellar stroke patients, we conducted a Pearson correlation analysis within the patient group. Besides reading-related measures, the analysis additionally included demographic variables (age, years of education), clinical variables (years since stroke occurrence, lesion size), and neuropsychological measures that showed significant group differences (Table 1): (9-Hole Peg Test, D–KEFS Color-Word Interference, WRMT Word Identification, and WRMT Word Attack).

To operationalize participants’ phonological decoding ability, we calculated a decoding deficit index by subtracting performance in the pseudoword condition from the word condition for both accuracy and latency measures in the LDT. The pseudoword-word differential in reading accuracy is often used as a criterion of phonological dyslexia and alexia (Beauvois & Derouesne, 1979; Castles & Coltheart, 1993; Tree, 2008). Thus, a higher decoding index value indicates greater difficulty in reading pseudowords relative to words.

**Figure 5:**
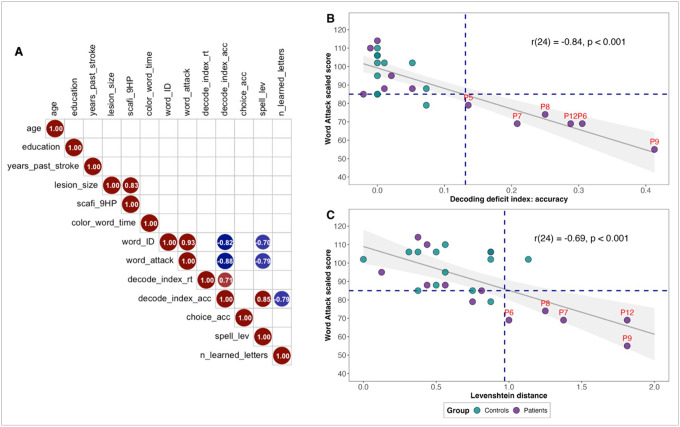
Individual differences in participant performance. A) Pearson’s correlations in the patient group (*ps* < 0.01). B) and C) Correlations across all groups between the WRMT Word Attack scores, the decoding deficit indices in the LDT, and the Levenshtein distances in the spelling test. In B and C, the horizontal blue line indicates below-average Word Attack scores; the vertical blue line marks scores 0.5 standard deviations above the mean. Individuals performing below the thresholds are labeled in red.

At an alpha level of 0.01, Pearson’s correlations revealed that WRMT Word Identification and Word Attack scores were negatively correlated with the decoding deficit index in the LDT (*r*(11) = − 4.83, *p* < 0.001, and *r*(11) = − 6.19, *p* < 0.001, respectively), as well as with Levenshtein distances in the spelling task (*r*(11) = − 3.29, *p* = 0.007, and *r*(11) = − 4.31, *p* = 0.001, respectively) (Fig. 5A). These findings indicate that lower scores on standardized WRMT subtests were associated with greater decoding difficulties and reduced spelling accuracy. Additionally, Levenshtein distances and decoding deficits were positively correlated (*r*(11) = 5.33, *p* < 0.001), suggesting that people with greater phonological decoding difficulties deviated from the target spellings of pseudowords to a greater extent. The decoding deficit index also negatively correlated with the number of letters learned in the artificial orthography training protocol (*r*(11) = − 4.24, *p* = 0.001), suggesting that greater decoding difficulties were also associated with poorer letter-sound learning outcomes. Lastly, lesion size was positively correlated with the 9-Hole Peg Test performance (*r*(11) = 4.93, *p* < 0.001), consistent with the ubiquitous accounts of the cerebellum’s role in motor functions.

### Evidence of phonological alexia

To identify individuals with phonological alexia, we triangulated participants’ performance across three measures: the WRMT Word Attack subtest (pseudoword reading), decoding deficit indices for reading accuracy in the LDT, and Levenshtein distances in the spelling task—measures that were significantly correlated (Fig. 5A). A cutoff score of 85 was applied for the Word Attack subtest, indicating below-average performance (Woodcock, 2011). For the decoding deficit indices and Levenshtein distances, we used a threshold of the mean plus 0.5 standard deviations. Based on these criteria, we identified five individuals (P6, P7, P8, P9, and P12), representing 38% of the patient group, who exhibited deficits across all three tasks (Figs. 5B, 5C). Notably, four of these individuals (P6, P7, P9, and P12) also failed to meet the threshold in the artificial orthography letter-sound mapping training (Fig. 4B). One participant (P5) had a borderline decoding deficit index (Fig. 5B) but did not exhibit impaired spelling performance (Fig. 5C) and was therefore not included in the alexia cohort.

### Lesion location analysis

**Figure 6:**
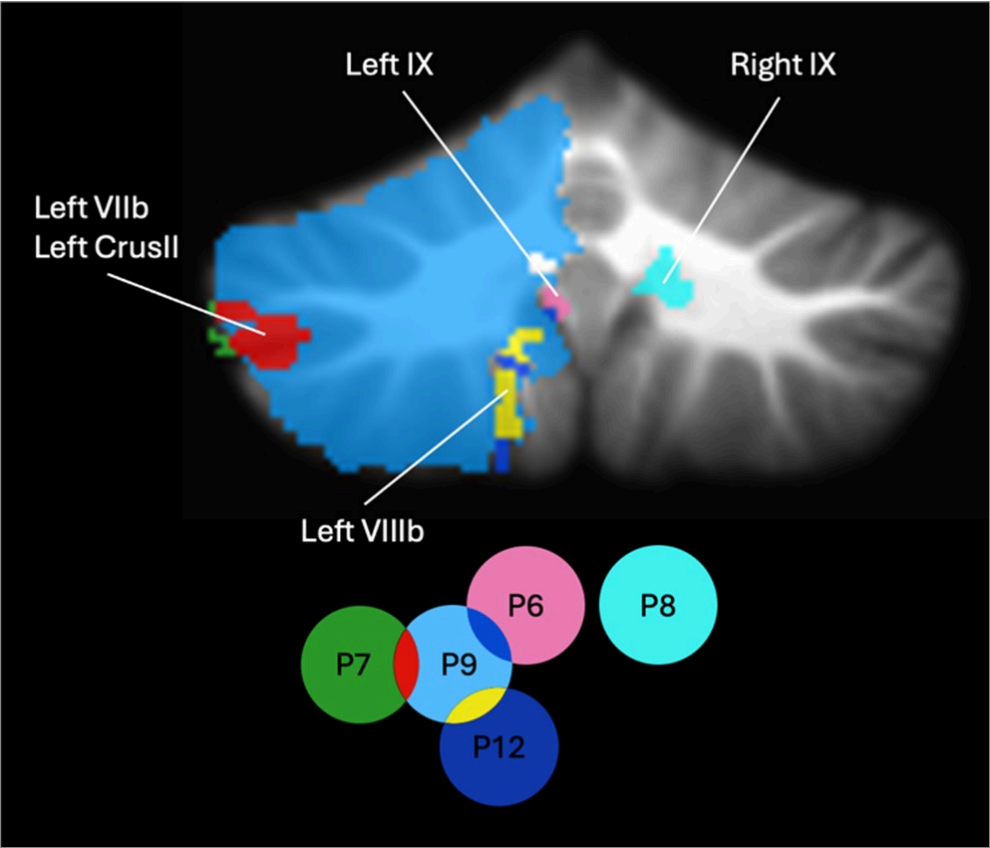
Overlay of cerebellar lesions in patients with phonological alexia, shown on the SUIT atlas template. Patient IDs are represented by color circles, with overlapping regions indicating shared lesion sites among patients. Lesions are color-coded by patient IDs.

To examine the relationship between reading deficits and lesion location, we mapped the lesions of the five patients who met the alexia criteria onto the SUIT atlas template (Diedrichsen, 2006) (Fig. 6). Lesion size and location varied considerably across patients. P9’s lesion encompassed nearly the entire left cerebellar hemisphere and overlapped with P7’s lesion in Left VIIb and Crus II, P12’s lesion in Left VIIIb, and P6’s lesion in Left IX. In contrast, P8 had a lesion in Right IX that did not overlap with any other lesions in the alexia group.

**Figure 7:**
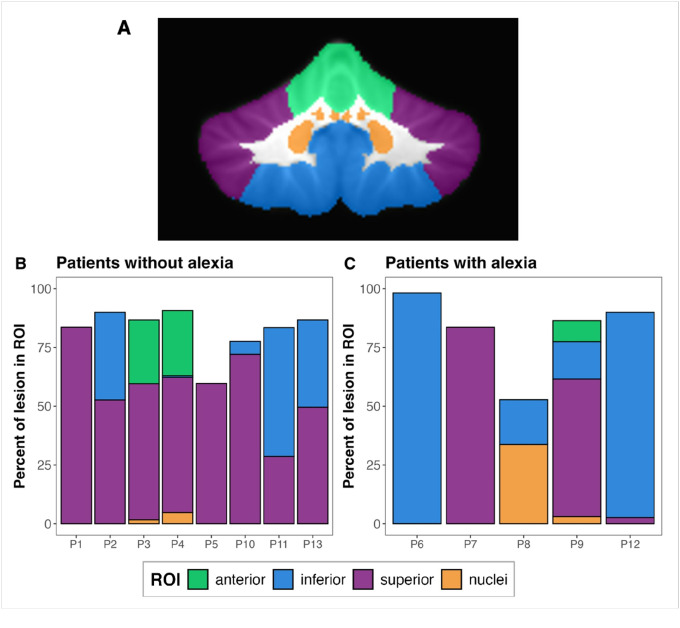
Region of interest (ROI) analysis results. A) Cerebellar partitioning into anterior, inferior, superior, and nuclear ROIs. B) Lesion distribution across ROIs in participants without alexia. C) Lesion distribution in participants with alexia.

To compare patterns of lesion location between individuals with and without alexia, we conducted a region of interest (ROI) analysis. Following Ravizza et al. (2006), we subdivided the cerebellum into four bilateral ROIs using the SUIT atlas: the anterior lobe (lobules I–V), superior lobe (lobules VI–VII and Crus I–II), inferior lobe (lobules VIII–X), and the cerebellar nuclei (dentate, interposed, fastigial) (Fig. 7A). For each patient, we calculated the percentage of lesion voxels within each ROI and compared the distribution between the two groups.

The results showed a heterogeneous distribution of lesions across ROIs, with no single region consistently associated with reading deficits (Figs. 7B, 7C). Additionally, there was no observed relationship between lesion lateralization and the presence of alexia. Although no clear association emerged between lesion location and alexia, all individuals with intact reading and decoding abilities had some lesion involvement in the superior ROI, suggesting that damage to this region alone may not be sufficient to impair reading abilities.

## Discussion

The present study investigated the impact of cerebellar stroke on both established, well-practiced reading skills and emergent literacy abilities through a combination of static assessment with English reading tasks and dynamic assessment with an artificial orthography learning paradigm. Comparisons between cerebellar stroke patients and control participants (matched on age, gender, race, ethnicity, and years of education) revealed that cerebellar damage is associated with diminished reading performance. Specifically, on static measures, individuals with cerebellar damage exhibited longer reaction times and lower accuracy rates on pseudoword reading in a lexical decision task. Reading deficits were also evidenced by reduced reading accuracy on the Word Identification and Word Attack subtests of the WRMT. In the dynamic assessment of emergent reading skills, individuals with cerebellar damage learned fewer letter-sound correspondences and achieved lower accuracy in reading the artificial orthography words relative to controls. These findings provide new evidence of a causal link between cerebellar damage and reading ability, extending earlier observations of acquired alexia following cerebellar stroke by Moretti et al. (2002). Furthermore, they highlight the cerebellum’s role not only in maintaining well-established reading processes but also in the ability to learn new letter-sound associations and apply phonological decoding rules to novel scripts.

### Acquired phonological alexia

A more nuanced analysis of stroke participants’ performance points towards the underlying phonological nature of reading difficulties, specifically, impairment of the process of connecting orthographic and phonological representations. This interpretation is supported by several observations. First, significant differences between stroke and control participants emerged only in the pseudoword condition but not in the real word condition of the LDT (Fig. 3A, 3B). Because pseudowords lack corresponding lexical representations, their decoding relies primarily on sublexical grapheme-phoneme conversion rather than whole-word lexical retrieval (Castles & Coltheart, 1993). While the precise computational mechanisms underlying this process remain debated (cf. the dual-route cascaded model (Coltheart et al., 2001) and the triangle model of reading (Plaut, 1997)), impaired pseudoword reading alongside relatively preserved real word reading is considered a hallmark symptom of phonological dyslexia/alexia (Beauvois & Derouesne, 1979; Friedman, 1995; Patterson & Ralph, 1999; Tree, 2008; Tree & Kay, 2006) that has been broadly linked to disruptions in the orthography-to-phonology conversion pathway (Stoodley & Stein, 2011; 2013; Tree & Kay, 2006). The pattern of deficits observed in our stroke participants suggests that cerebellar damage selectively impairs this mechanism while leaving lexical retrieval relatively intact.

Beyond established reading skills, this impairment extended to emergent literacy abilities, as demonstrated by stroke participants’ difficulty to learn novel letter-sound correspondences in the artificial orthography training paradigm, with only six out of 13 people meeting the learning threshold (Fig. 4B). Even among those who met the threshold, performance on the artificial orthography word reading task remained lower than that of controls (Fig. 4C). Importantly, this deficit cannot be attributed to a generalized phonological impairment, as has been proposed by some theories of phonological dyslexia (Harm & Seidenberg, 2001; Patterson & Marcel, 1992), because our stroke participants performed within the normative range on rapid letter naming (CTOPP) (Wagner et al., 2013) and verbal working memory (RAVLT) (Rey, 1983) tests, suggesting intact phonological storage and retrieval. Thus, this selective deficit supports the interpretation of a specific disruption in phonological decoding rather than a generalized phonological impairment.

An alternative explanation for the reduced reading performance in stroke participants could stem from impaired orthographic processing, i.e., difficulties learning the visual form of new words. Indeed, reading problems have been linked to deficits in orthographic encoding, particularly in cases where readers struggle to store, retrieve, or manipulate novel word forms in memory (Badian, 2005; Gustafson, Ferreira, & Rönnberg, 2007; Paul, Baca, & Fischer-Baum, 2022). However, our findings do not support this interpretation. When tested on their ability to recognize and spell pseudowords introduced two hours earlier, stroke participants performed comparably to controls, suggesting preserved orthographic encoding and retrieval. If their reading difficulties were driven by orthographic deficits, we would expect impaired encoding of novel word forms, yet this was not observed (Fig. 3C, 3D). This further reinforces the conclusion that the primary deficit lies in phonological decoding rather than orthographic representation.

### Alexia symptom severity and lesion location

Our cohort of cerebellar stroke patients exhibited considerable variability in their established and emergent reading skills. Some patients experienced marked difficulty in decoding unfamiliar word forms (pseudowords) and forming new letter-sound associations, while others exhibited relatively preserved abilities. Using a pseudoword-word differential approach to quantify phonological alexia, we identified five of the 13 stroke participants who met the diagnostic criteria (Fig. 5B, 5C), echoing previous reports of substantial variability in cognitive outcomes following cerebellar damage (Timmann & Daum, 2010). Notably, the decoding deficit index was significantly correlated with established reading measures such as the WRMT Word Identification and Word Attack scores, as well as with spelling accuracy and the number of learned letter-sound correspondences (Fig. 5A). These results suggest a common underlying deficit affecting both established reading processes and novel letter-sound learning.

Lesion mapping analysis additionally revealed substantial heterogeneity in lesion locations among our patients. Importantly, there was no clear relationship between lesion location and the presence of alexia (Fig. 6), suggesting that phonological alexia likely does not arise from damage to a single cerebellar lobule, but rather from disruption of a distributed network of regions. This finding aligns with recent evidence that lobular boundaries do not necessarily reflect boundaries in functional specialization (Nettekoven et al., 2024). Furthermore, although reading-related processes are often associated primarily with right cerebellar activation (e.g., Stoodley, 2012; Li et al., 2021, 2022; Li, Yuan, Luo, & Tao, 2022), four of our patients with phonological alexia had lesions in the left cerebellum, indicating that both hemispheres contribute to decoding processes, particularly in the context of stroke-induced lesions. Finally, our ROI analysis further revealed that the location of cerebellar damage does not yield a predictable pattern of reading impairment. Even when the superior cerebellum—typically associated with higher-order cognitive functions (Schmahmann, 2019; Stoodley & Schmahmann, 2010)—was affected, not all patients exhibited symptoms of phonological alexia (Fig. 7). These observations imply that compensatory mechanisms or the distributed nature of cerebro-cerebellar connections may mitigate the impact of focal damage on reading performance.

### What is the role of the cerebellum in phonological decoding?

The ability to read novel words—whether in a familiar orthography (e.g., pseudowords) or an unfamiliar one (e.g., a new writing system)—relies heavily on phonological decoding (Ehri, 2005; Rozin & Gleitman, 1977; Share, 1995; 2008). This process requires readers to break words apart into understandable units and then put them back together to construct meaning. Phonological decoding engages a series of operations, including letter identification, syllabification, integration of orthographic and phonological codes, and maintenance of phonological information in working memory until a coherent match is achieved. The cerebellum may be involved in scaffolding several of these operations through its participation in the phonological loop—a core component of the working memory (Baddeley, 2003). Indeed, several studies have implicated the cerebellum in phonological processes, suggesting that cerebellar damage impacts the efficiency of the phonological loop, particularly its subvocal rehearsal component and articulatory monitoring function (Ackermann, Mathiak, & Riecker, 2007; Ben-Yehudah & Fiez, 2008; Fawcett, Nicolson, & Dean, 1996). These functions are especially critical when reading unfamiliar words or acquiring new orthographic-phonological mappings, as they require increased articulatory effort and error monitoring. Accordingly, cerebellar damage may impair reading performance in tasks that depend on these abilities, such as pseudoword reading and decoding in artificial orthographies.

Beyond its direct role in phonological processing, the cerebellum may contribute indirectly to phonological decoding by supporting broader cognitive control processes such as attentional regulation, inhibitory control, error detection, memory maintenance, and task updating. Multiple studies have documented the cerebellum’s involvement in cognitive control and executive functions (e.g., Bellebaum & Daum, 2007; Clark et al., 2021; D’Mello, Gabrieli, & Nee, 2020; Schmahmann, 2019), some of which are particularly relevant to phonological decoding. For example, decoding a novel item like ‘bleez’ requires selecting the correct pronunciation while inhibiting competing phonological (e.g., ‘please’, ‘fleas’) and orthographic (e.g., ‘bees’, ‘bleed’) alternatives. Likewise, learning new letter-sound associations entails focusing on relevant phonological cues while filtering out irrelevant visual or auditory information, and suppressing interference from previously learned letter-sound associations in the native language. These selection, inhibition, and fine-tuning functions have been linked to the cerebellum (e.g., Mannarelli et al., 2020; Mostofsky et al., 2003; Palacios, Houghton, & Chadderton, 2021; Picazio et al., 2020). Accordingly, damage to the cerebellum may impair these mechanisms. Consistent with this view, patients with cerebellar damage in our study exhibited diminished performance on the D-KEFS Color-Word Interference task, indicating compromised selection-inhibition control relative to healthy controls.

Furthermore, deficits in decoding unfamiliar words and forming new letter-sound associations observed in some cerebellar stroke patients may reflect general impairments in implicit learning. Clinical studies have demonstrated that cerebellar patients often exhibit deficits in both associative (Timmann et al., 2002, 2004) and procedural learning (Gomez-Beldarrain et al., 1998; Molinari et al., 1997). Similarly, patterns of impaired implicit learning with preserved explicit learning have been documented in dyslexic children (Stoodley et al., 2008; Vicari et al., 2005). The cerebellum’s role in learning is further supported by evidence showing that its activity is heightened during the initial phases of learning and diminishes as skills become more automatic (AUTHORS; Raichle et al., 1994; Van Mier & Petersen, 2002). Given that implicit learning and skill automatization are critical during the initial phases of reading acquisition, damage to the cerebellum may underlie difficulties with emergent reading skills, such as establishing new letter-sound associations, adapting to novel writing systems, and automatizing a new articulatory sequence that corresponds to the grapheme-phoneme string. This can also explain compromised pseudoword reading alongside a relatively intact real word reading, because the novel nature of pseudowords necessitates formation of new decoding routines and experience-dependent adjustments to achieve accurate pronunciation.

To summarize, cerebellar damage can affect multiple aspects of phonological decoding, ranging from deficits in subvocal rehearsal to broader learning challenges. However, the variability in lesion size and location among patients complicates definitive conclusions regarding the cerebellum’s specific contributions to phonological decoding. This heterogeneity highlights the potential influence of individual factors—including patient history, post-stroke rehabilitation, compensatory strategies, and possibly pre-morbid reading abilities—on the severity of reading impairments following cerebellar damage. Future research should further examine these variables to clarify their roles in post-stroke reading deficits.

## Conclusion

The present study provides novel insights into the cerebellum’s role in reading by integrating static and dynamic assessments of established and emergent reading abilities in patients with cerebellar stroke. The results provide converging evidence that cerebellar damage disrupts phonological decoding processes, affecting both established reading skills in a familiar orthography and the acquisition of novel orthographic-phonological mappings. The observed impairments are not attributable to general deficits in phonological awareness, working memory, or orthographic processing, but rather to a specific disruption in sublexical decoding mechanisms, consistent with acquired phonological alexia. Variability in lesion location and behavioral symptoms underscores the complexity of cerebellar contributions to reading and highlights the likely involvement of a distributed network. These results not only expand our understanding of the cerebellum’s role in reading but also have clinical implications for diagnosis and rehabilitation of reading disorders following cerebellar stroke. Future research should aim to delineate the functional contributions of distinct cerebellar regions and investigate individual variability in post-stroke recovery trajectories.

## Supplementary Material

Supplementary Files

This is a list of supplementary files associated with this preprint. Click to download.

Appendix.docx

## Figures and Tables

**Table 1:** Neuropsychological outcomes for the cerebellar stroke patients (n = 13) and the control participants (n = 13).

Function	Measure	Units	Mean (SD)		*t* value	*p* value
			Patients	Controls		
Motor	8-Meter Walking	seconds	6.1 (1.3)	5.8 (1.7)	−0.45	0.66
	9-Hole Peg	seconds	**32.1 (10.7)**	**24.1 (4)**	−**2.5**	**0.02**
	Finger Tapping	average count	34.2 (9.1)	34.6 (5.9)	0.11	0.91
	SARA	total score	1.8 (2)	0.9 (1.5)	−1.33	0.20
Cognitive	MoCA	total score (max 30)	24.8 (4)	27.1 (1.9)	1.93	0.07
	RAVLT	% correct	55.3 (10)	55.6 (11.2)	0.07	0.94
	D–KEFS Color-Word Interference [time]	scaled score	**9 (3.1)**	**11.9 (2.3)**	**2.71**	**0.01**
	D–KEFS Color-Word Interference [error]	count	5.7 (7.4)	1.4 (2.3)	−2.01	0.06
Reading	WRMT Word ID	standard score	**93 (15.4)**	**105.5 (9.4)**	**2.51**	**0.02**
	WRMT Word Attack	standard score	**84.7 (18)**	**97.8 (10.2)**	**2.3**	**0.03**
	CTOPP Rapid Letter Naming [time]	scaled score	6.6 (3.1)	8.4 (2.9)	1.5	0.15
	CTOPP Rapid Letter Naming [error]	count	0	0	N/A	N/A

Note. Bold font marks significant comparisons at alpha 0.05. For standard and scaled scores, greater values mean better performance.
